# Refractory Thrombotic Thrombocytopenic Purpura in a Patient With Triple X Syndrome

**DOI:** 10.7759/cureus.67631

**Published:** 2024-08-23

**Authors:** Pedro Arthur da Rocha Ribas, Julia Ghiraldi, Giovanna Gugelmin, Lucas Wagner Gortz, Mauricio de Carvalho, Gustavo Lenci Marques

**Affiliations:** 1 Department of Internal Medicine, Clinical Hospital Complex of the Federal University of Paraná, Curitiba, BRA; 2 Department of Internal Medicine, School of Medicine, Federal University of Paraná, Curitiba, BRA; 3 Department of Internal Medicine, Pontifical Catholic University of Paraná, Curitiba, BRA

**Keywords:** chromosomal abnormality, adamts13, vincristine, triple x syndrome, thrombotic thrombocytopenic purpura

## Abstract

Clinical manifestations of triple X syndrome (karyotype 47, XXX) can include autoimmune diseases. We describe the occurrence of acquired thrombotic thrombocytopenic purpura (TTP), an autoimmune condition, refractory to plasmapheresis and rituximab in a patient with triple X syndrome who required vincristine administration for disease remission. To our knowledge, this rare coexistence is the first of its kind reported in Brazil.

## Introduction

Triple X syndrome (karyotype 47, XXX) is the most common chromosomal abnormality among women, with a prevalence of approximately 1 in every 1,000 female births. However, due to the fact that many women affected by this syndrome have no symptoms or are oligosymptomatic, it is estimated that only 10% of cases are actually diagnosed. When present, some of the clinical manifestations of this syndrome include low muscle mass, cognitive deficit, epilepsy, genitourinary and cardiac malformations, abnormalities in the reproductive system, autoimmune diseases, and greater stature when compared to the average of their respective families. The first report of triple X syndrome used the term "super females" to describe the karyotype [1,2].

Thrombotic thrombocytopenic purpura (TTP) is a medical emergency characterized by thrombocytopenia associated with microangiopathic hemolytic anemia and organ damage due to visceral ischemia, resulting from deficiency of the enzyme ADAMTS13 (a disintegrin and metalloproteinase with thrombospondin type 1 motif, member 13); there should be strong suspicion for this disorder due to its high lethality rate in case of delay in the initiation of therapy [3]. Although its clinical picture is classically described as the presence of a pentad (fever, neurological changes, acute renal dysfunction, anemia, and thrombocytopenia), TTP can occur with different clinical manifestations, as all components of the pentad are present in only a minority of cases. Additionally, the limited availability of ADAMTS13 testing in some cases contributes to the difficulty in diagnosing this condition [4].

## Case presentation

A 42-year-old Black female patient was diagnosed with triple X syndrome (by karyotyping through bone marrow aspirate) and TTP in December 2020 during hospitalization due to metrorrhagia, anemia with schizocytes in peripheral blood, and thrombocytopenia (Table 1). She had no other symptoms, apart from denying the use of previous medications. The diagnostic suspicion of TTP was raised due to her high PLASMIC score of 7, comprising the following findings: platelets, 29,000 mm3; reticulocytes, 9%; absence of active cancer; absence of solid organ transplantation; mean corpuscular volume 88 fl; INR 1.2; creatinine 0.8 mg/dl. Other diseases, like systemic lupus erythematosus (SLE), were investigated and ruled out. Due to these findings, treatment with plasmapheresis and rituximab was promptly initiated, but due to the unsatisfactory response, it was necessary to administer vincristine. After this, the patient showed clinical and laboratory improvement, undergoing 18 sessions of plasmapheresis, two doses of rituximab, and four doses of vincristine.

**Table 1:** Admission laboratory tests (December 2020)

Parameters	Value	Reference Range
Hemoglobin	8.6 g/dl	12.3-15.3 g/dl
Hematocrit	23%	35.9-44.6%
Mean corpuscular volume	88 fl	80-96 fl
Reticulocytes	9%	0.5-1.5%
Haptoglobin	1.0 mg/dl	30-200 mg/dl
Platelets	29,000 /mm^3^	150,000-450,000 /mm^3^
Total bilirubin	3.20 mg/dl	0.3-1.0 mg/dl
Indirect bilirubin	1.93 mg/dl	0.1-1.0 mg/dl
Lactate dehydrogenase	1,902 UI/L	100-190 UI/L
Creatinine	0.80 mg/dl	≤ 1.10 mg/dl
Estimated glomerular filtration rate	97 ml/min/1.73m^2^	90-120 ml/min/1.73m^2^
International normalized ratio (INR)	1.19	0.9-1.2
Prothrombin time (PT)	13.1 seconds	10-14 seconds
Activated partial thromboplastin time (aPTT)	27.1 seconds	20-32 seconds
Fibrinogen	213 mg/dl	150-400 mg/dl
D-dimer	3.95 mg/L	< 0.5 mg/L

She had the first relapse of the disease in March 2023, when she required seven sessions of plasmapheresis and two doses of rituximab for remission of the disease. She was discharged from the hospital 10 days after admission without administration of vincristine on that occasion. The patient developed an exacerbation of the disease with vaginal bleeding, headache, and thrombocytopenia of 6,000 platelets/mm3 less than 30 days after her hospital discharge, requiring rehospitalization; this time requiring 20 sessions of plasmapheresis in addition to the administration of rituximab and vincristine due to therapeutic refractoriness. After these hospitalizations, the patient was scheduled to follow up at the hematology outpatient department.

In January 2024, in a routine outpatient appointment with the hematology department, the patient reported intense headaches that started five days before the appointment but denied bleeding or other symptoms. Follow-up laboratory tests (Table 2) showed macrocytic anemia, thrombocytopenia, and altered hemolytic results as well as schizocytes in the peripheral blood. In view of the evidence of a new relapse of TTP, the patient was referred for hospitalization again; she was initially admitted to the ICU to stabilize her clinical condition and was subsequently transferred to a ward. Her treatment initially involved plasmapheresis sessions and the administration of methylprednisolone and rituximab. However, due to the lack of satisfactory response, she also required the administration of vincristine for remission of the disease. She was discharged in February 2024.

**Table 2:** Follow-up laboratory tests (January 2024)

Parameters	Value	Reference Range
Hemoglobin	8.7 g/dl	12.3-15.3 g/dl
Hematocrit	26%	35.9-44.6%
Mean corpuscular volume	105 fl	80-96 fl
Reticulocytes	8%	0.5-1.5%
White blood cells	6,370 /mm^3^	4,400-11,000 /mm^3^
Platelets	23,400 /mm^3^	150,000-450,000 /mm^3^
Total bilirubin	2.34 mg/dl	0.3-1.0 mg/dl
Indirect bilirubin	1.69 mg/dl	0.1-1.0 mg/dl
Lactate dehydrogenase	724 UI/L	100-190 UI/L
Creatinine	0.66 mg/dl	≤ 1.10 mg/dl
Estimated glomerular filtration rate	112 ml/min/1.73m^2^	90-120 ml/min/1.73m^2^

## Discussion

Autoimmune diseases, including systemic lupus erythematosus (SLE), Sjogren's syndrome (SS), and thyroid disease have been associated with triple X syndrome [1]. In 1990, according to a case report by Itoh et al., the presence of triple X syndrome was identified in a 69-year-old patient with acquired TTP, an autoimmune condition, but when the tests were repeated, chromosomal alterations were no longer evidenced, and the hypothesis of transient chromosomal aberrations and TTP being related to viral infections was raised [5]. Nonetheless, after a literature search, no other case reporting the coexistence between triple X syndrome and TTP was found.

TTP occurs when there is a severe deficiency of the enzyme ADAMTS13 (activity typically < 10%). Deficiency of this enzyme can be hereditary (inherited mutations in the ADAMTS13) or acquired (presence of autoantibodies against ADAMTS13), occurring predominantly in young women [3,6]. Acquired (autoimmune) TTP is developed due to the advent of IgG autoantibodies against ADAMTS13, leading to the destruction of this enzyme and its deficiency [7,8]. The ADAMTS13 is responsible for cleaving the large von Willebrand factor (VWF) multimers into smaller multimers. When deficient, there is an accumulation of larger VWF multimers in the plasma, which are more efficient in platelet binding and activation than smaller multimers. This triggers microvascular thrombosis, resulting in thrombocytopenia due to platelet consumption and microangiopathic hemolytic anemia, which manifests through the presence of schizocytes in the peripheral blood [9,10]. Therefore, in acquired TTP, the pathophysiological mechanism of the disease is autoimmune, although the destruction of platelets does not occur directly through the action of autoantibodies on these cells, but rather through consumption in the microvasculature.

Black individuals have a higher risk of developing autoimmune TTP since studies have shown that they are susceptible to reduced frequency of a protective allele, known as HLA DRB1*04, which makes them more prone to developing autoantibodies against ADAMTS13, contributing to the greater number of patients of this race in TTP registries [11]. Autoimmune TTP is also more common in adult, female, and Black patients, like in our present case, while the congenital form of TTP is rarer and usually manifests itself in childhood [8].

The classic pentad manifestations include fever, neurological changes, acute renal dysfunction, anemia, and thrombocytopenia, but the presence of all components of the pentad at the same time occurs only in a minority of cases. Patients may also present with other symptoms, such as bleeding, most commonly mucocutaneous (severe bleeding is uncommon), due to thrombocytopenia, and abdominal pain, due to thrombosis and intestinal ischemia [3,6]. In the present case report, our patient presented only two symptoms of the classic pentad of TTP, which were hemolytic anemia and thrombocytopenia.

The management of TTP is a challenge, since the diagnostic criteria are not precise, which can result in uncertainty about the ideal time to start treatment [6]. The PLASMIC score has been used to contribute to the diagnosis of TTP, although it does not confirm or exclude this condition. It is calculated using clinical and laboratory findings, predicting the probability of reduced ADAMTS13 activity. The parameters evaluated in the PLASMIC score are described in Table 3, where each of these parameters, when present, is equivalent to 1 point. The higher the score, the more likely the diagnosis of TTP. A score ≥ 6 predicts an activity of ADAMTS13 ≤ 10% with a sensitivity of approximately 90%, leading to high diagnostic suspicion for TTP and indicating early initiation of its treatment [12]. The ease of calculating the PLASMIC score makes it a valuable clinical tool when testing for ADAMTS13 measurement is not available and urgent therapy is required [13]. In our patient, ADAMTS13 activity was not measured due to the limited availability of ADAMTS13 testing. The initial diagnosis of TTP is based on clinical history, physical examination, laboratory exams, and the presence of schizocytes in the peripheral blood [14].

**Table 3:** PLASMIC Score

Parameters	Points
Thrombocytopenia < 30,000/mm^3^	1
Hemolysis (reticulocytes > 2.5%, undetectable haptoglobin or indirect bilirubin > 2 mg/dl)	1
Absence of active cancer	1
Absence of solid organ transplantation or hematopoietic stem cells	1
Mean corpuscular volume < 90 fl	1
INR < 1.5	1
Creatinine < 2 mg/dl	1

A differential diagnosis that can be established with TTP is hemolytic uremic syndrome (HUS), characterized by the triad of anemia, thrombocytopenia, and acute renal dysfunction. However, in HUS, acute renal dysfunction is of greater significance and is typically preceded by diarrhea caused by *Escherichia coli*, in addition to being more common in children. Our patient did not present with acute renal dysfunction and there was no history of diarrhea on any of the occasions she was admitted to the hospital [7].

Regarding the therapeutic approach, plasmapheresis is the first line of treatment and should be started early, as soon as there is a high diagnostic suspicion for TTP since delays in initiating plasmapheresis are an independent predictor of treatment failure [15]. The beginning of treatment should not be delayed pending results of ADAMTS13 [16]. Plasmapheresis works by removing autoantibodies and large VWF multimers from the plasma and should be performed daily for at least two days until the platelet count stabilizes. In cases of refractory TTP, treatment should be intensified, and up to two daily sessions of plasmapheresis can be performed [8,17]. Refractory TTP is defined by the presence of clinical deterioration or non-improvement in platelet count or lactate dehydrogenase after five plasmapheresis sessions [18]. The incidence of patients who do not respond to plasmapheresis is up to 42% [14].

Among some of the other therapeutic options, we can list the administration of corticosteroids, which has a low efficacy [17], rituximab, which induces remission of the disease and prevents future relapses [8], and caplacizumab, a monoclonal antibody that binds to VWF and prevents its action on platelets. Caplacizumab use led to favorable outcomes such as increasing platelet count in a shorter period, a lower recurrence rate, and disease remission requiring fewer plasmapheresis sessions [19]. According to guidelines, TTP relapses should be managed with the administration of corticosteroids and rituximab, in addition to plasmapheresis [20].

Studies have confirmed the efficacy and safety of rituximab for TTP [15]. A meta-analysis showed that rituximab, when administered in the acute phase of the disease, results in lower rates of relapse and mortality [21]. Rituximab also appears to be effective in patients with refractory TTP or poor response to plasmapheresis [8]. However, when race is taken into account, another study showed that rituximab reduced the number of relapses in White patients, but not in Black patients. Thus, Black patients may need closer monitoring, early re-treatment, and alternative immunosuppression [22].

A study involving 15 patients evaluated the use of vincristine as a therapeutic option in cases of refractory or relapsed TTP, showing remission of the disease in 87% of the patients undergoing this treatment, with good tolerance of the medication. Among the side effects of vincristine use are sensory neuropathy, muscle weakness, paralytic ileus, leukopenia, and alopecia [23]. Several other studies have also demonstrated evidence of the efficacy of vincristine in the treatment of TTP [24, 25]. In the present case report, the patient was treated with plasmapheresis, methylprednisolone, and rituximab, but due to the lack of satisfactory response, it was necessary to use vincristine as a complementary therapy, with no side effects being observed. 

Historically, patients with TTP had a mortality rate of around 90%, but with the improvement of therapy, this rate has dropped to approximately 20% in recent years [3]. The patient in the present report remains in remission and is under outpatient follow-up.

## Conclusions

Studies have shown that triple X syndrome may be associated with autoimmune diseases. In the reported case, we describe a patient with acquired TTP, an autoimmune disease, and triple X syndrome refractory to standard treatment. However, studies are needed to assess whether there is a correlation between triple X syndrome and the development of TTP with a higher probability of therapeutic refractoriness.
